# Supplementary material for: Stability of Porous Polymeric Membranes in Amine Solvents for Membrane Contactor Applications
Source: Membranes (Basel). 2023 May 23;13(6):544. doi: 10.3390/membranes13060544 (PMC10301957; doi:10.3390/membranes13060544)
Supplement: Supplementary file 1 [file membranes-13-00544-s001.zip › membranes-2385631-supplementary.pdf]

membranes

Supplementary Materials

Stability of Porous Polymeric Membranes in Amine Solvents for Membrane Contactor Applications

Denis Kalmykov *, Sergey Shirokikh, Evgenia A. Grushevenko, Sergey A. Legkov, Galina N. Bondarenko, Tatyana S. Anokhina, Sergey Molchanov and Stepan D. Bazhenov *

A.V. Topchiev Institute of Petrochemical Synthesis RAS, 29 Leninsky Prospekt, 119991 Moscow, Russia
* Correspondence: denis.kalmykov@ips.ac.ru (D.K.); sbazhenov@ips.ac.ru (S.D.B.); Tel.: +7-495-647-59-27 (ext. 2-02) (S.D.B.)

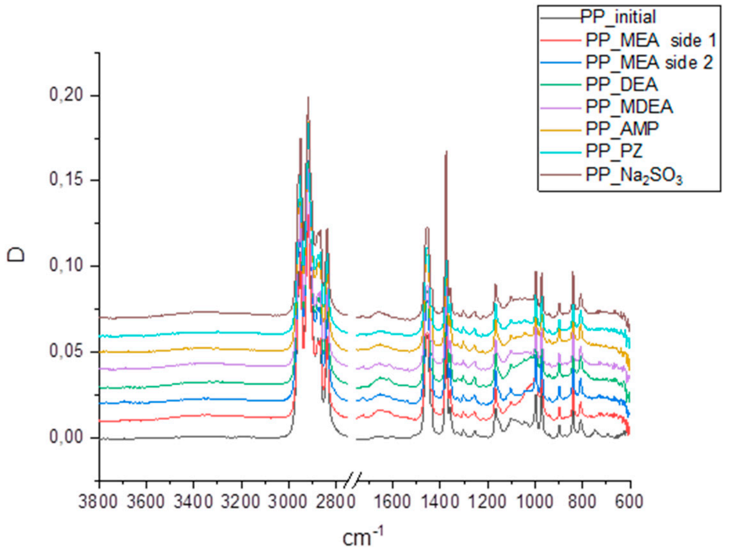

Figure S1. FTIR spectra of PP membrane samples before and after exposure to model solutions.

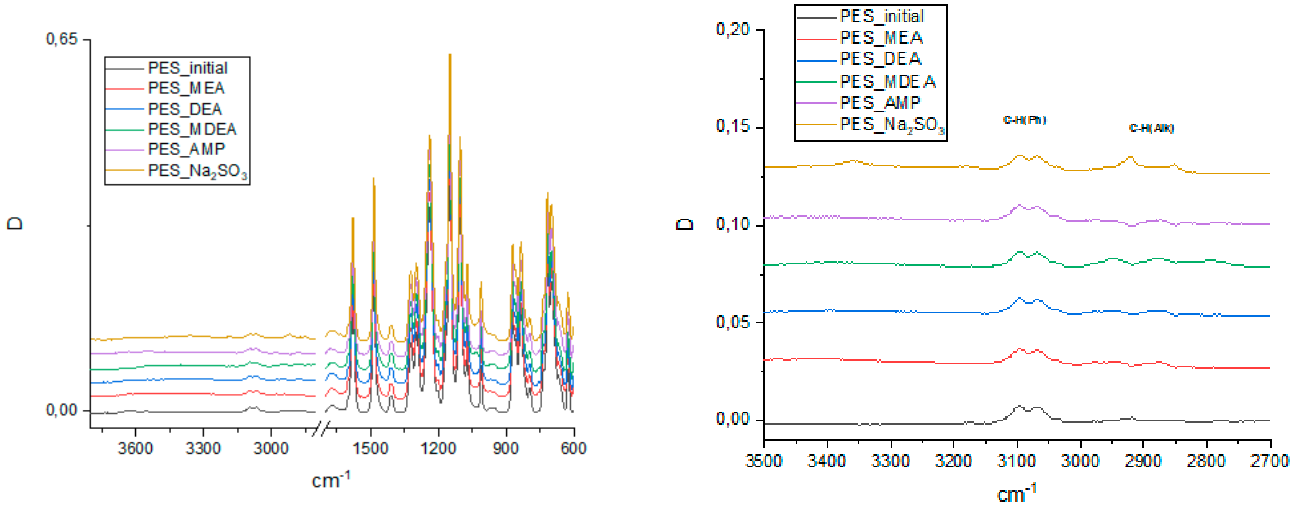

(**a**) (**b**)

Figure S2. FTIR spectra of PES membrane samples before and after exposure to model solutions (**a**), detailed spectra fragments (**b**).

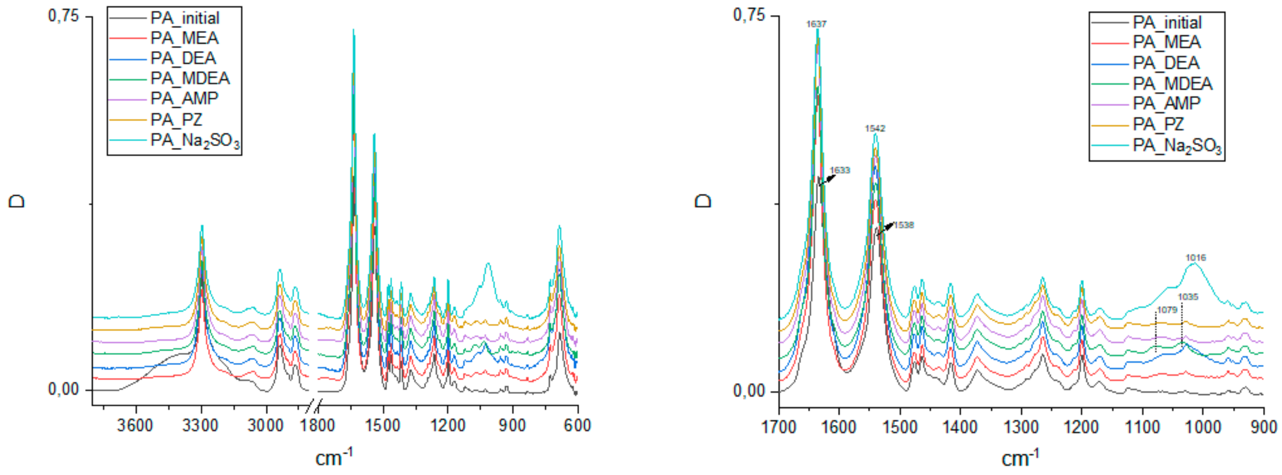

(**a**) (**b**)

Figure S3. FTIR spectra of PA membrane samples before and after exposure to model solutions (**a**), detailed spectra fragments (**b**).

Table S1. AFM images of PVDF membranes before and after exposure to model solutions.

Solution	20×20 μm	ΔZ, μm	10×10 μm	ΔZ, μm
**Initial**	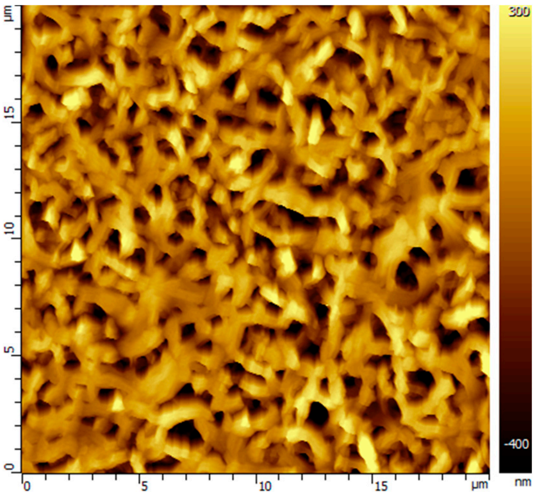	0,7	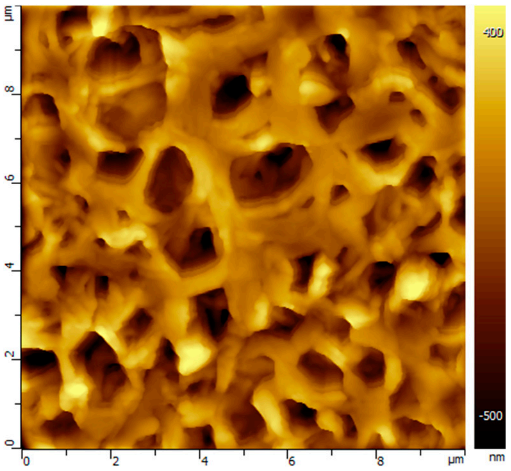	0,9
**MEA**	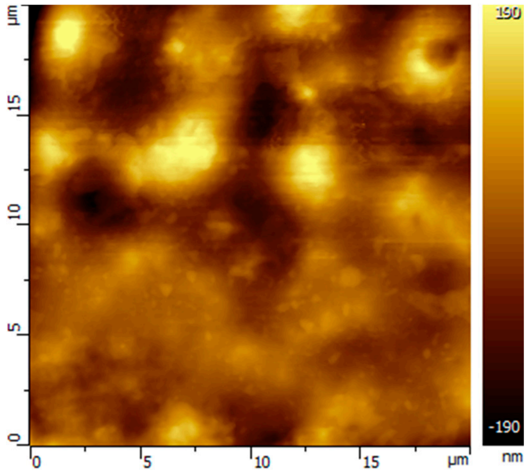	0,38	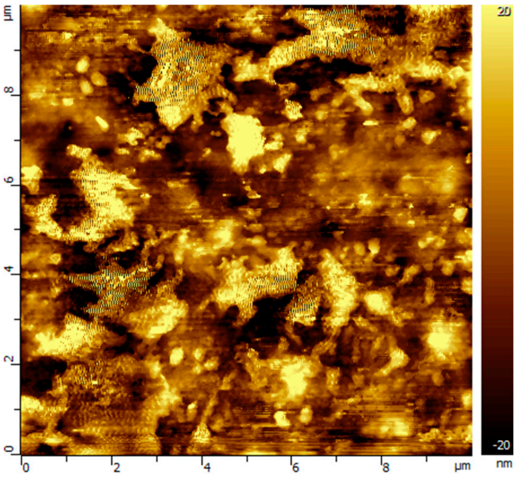	0,04
**DEA**	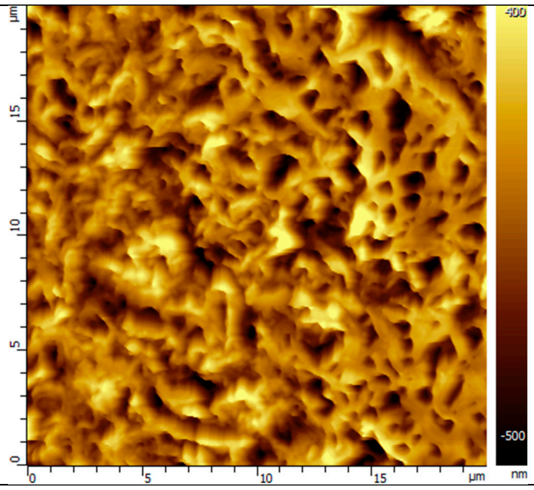	0,9	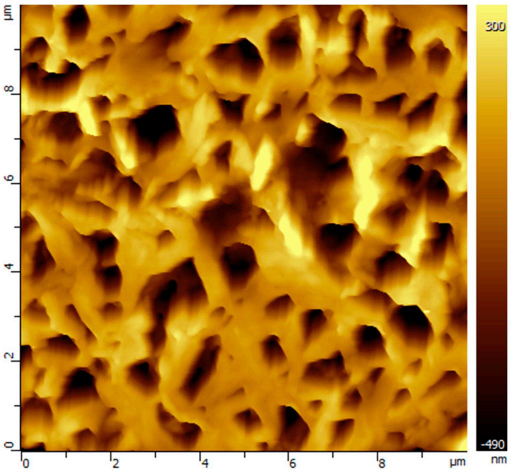	0,79

**MDEA**	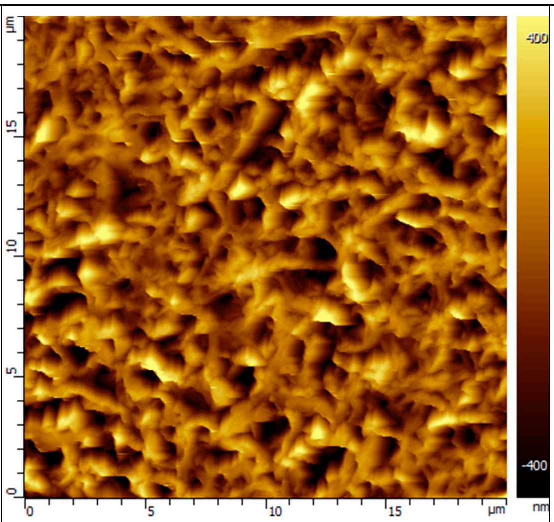	0,8	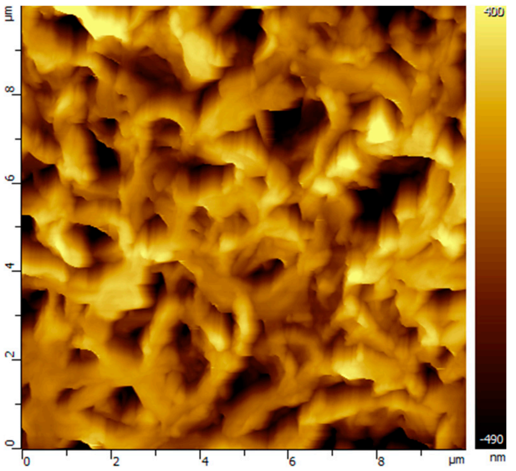	0,89
**AMP**	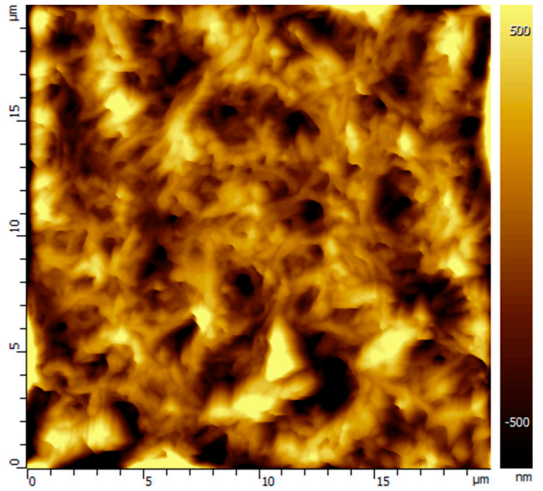	1,0	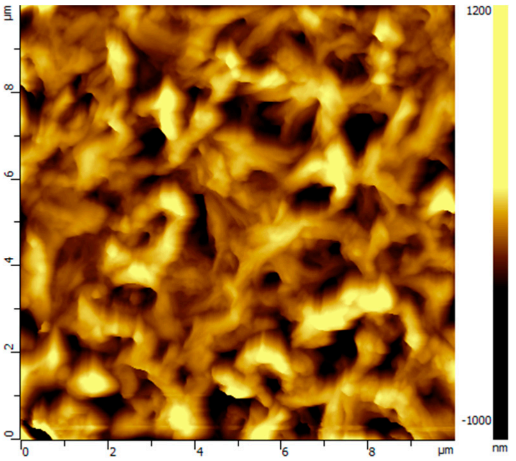	2,2
**PZ**	-			
**Na_2SO_3**	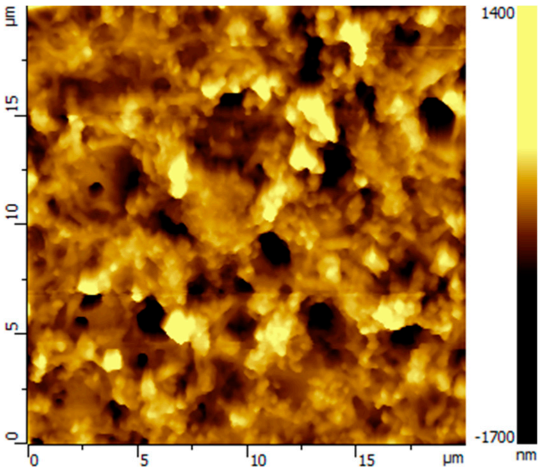	3,1	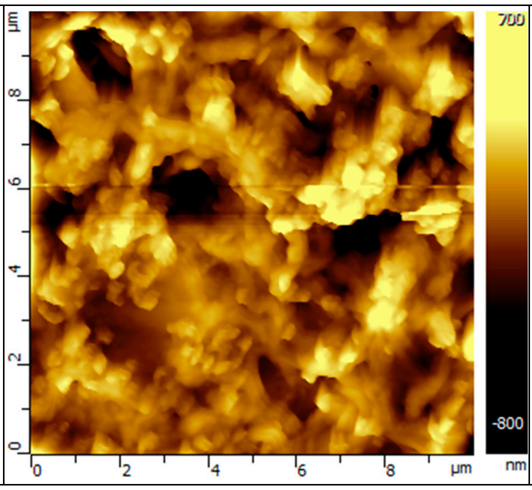	1,5

Table S2. AFM images of PTFE membranes before and after exposure to model solutions.

Solution	60×60 μm	ΔZ, μm	20×20 μm	ΔZ, μm
**Initial**	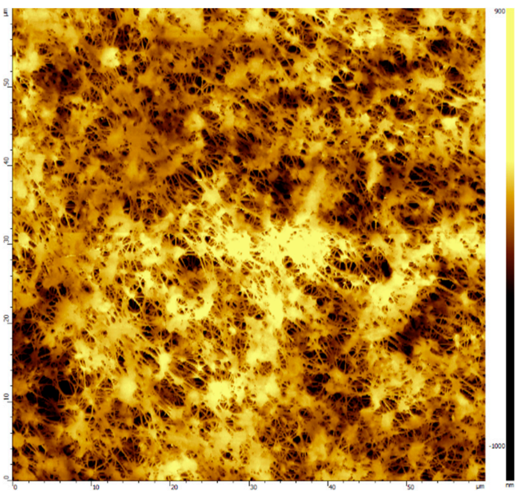	1,9	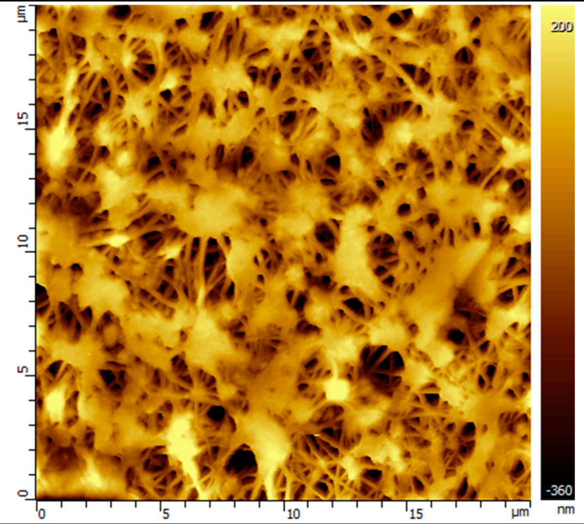	0,56
**MEA**	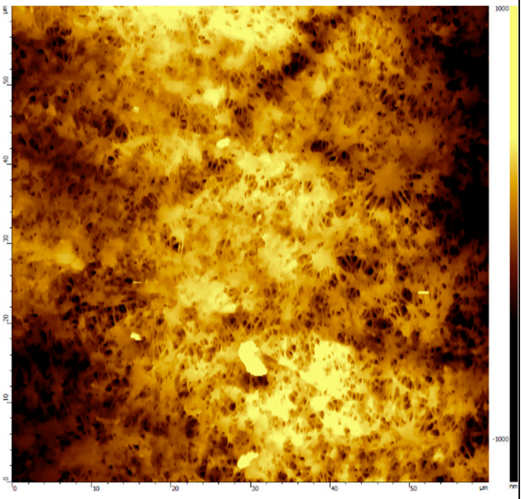	2,0	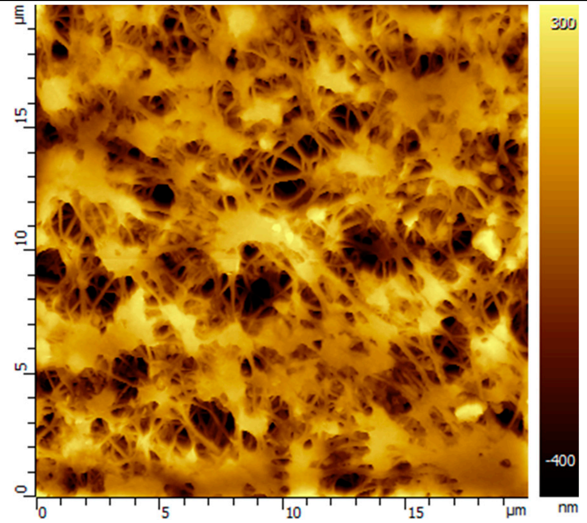	1,3
**DEA**	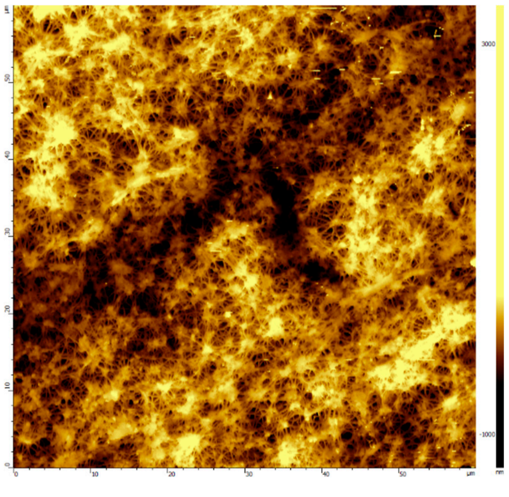	4,0	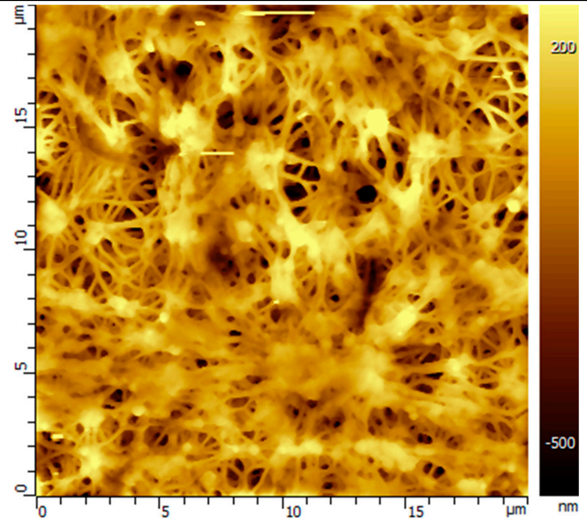	0,7

**MDEA**	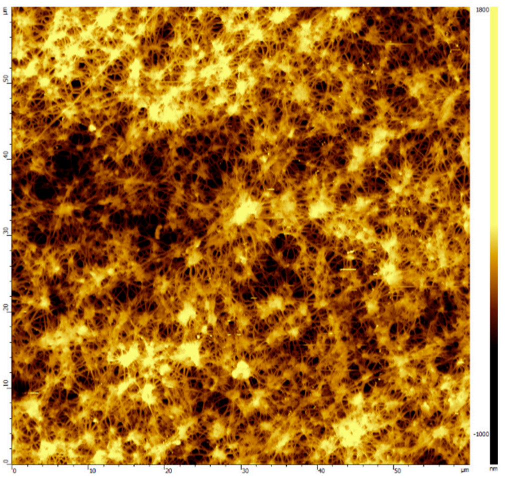	2,8	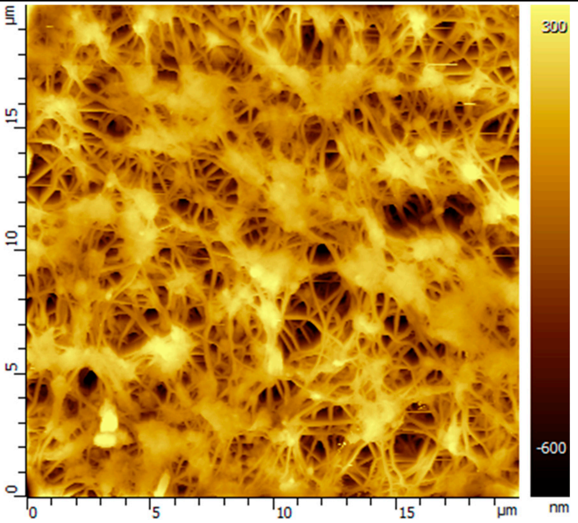	0,9
**AMP**	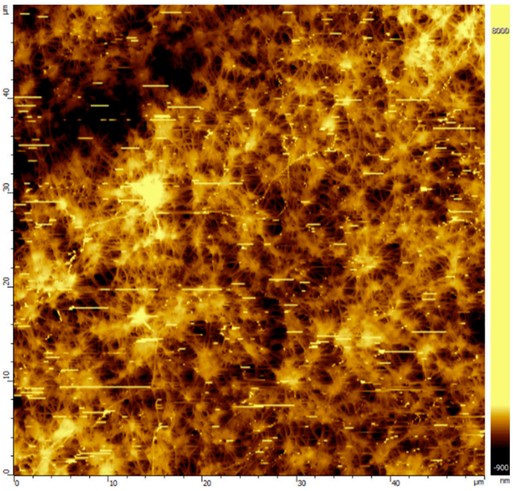	8,9	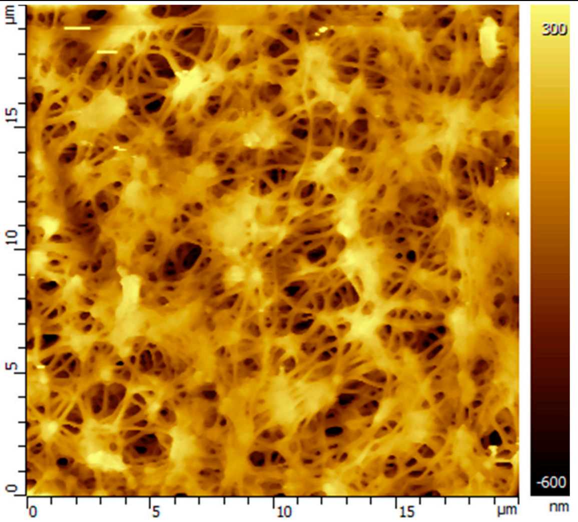	0,9
**PZ**	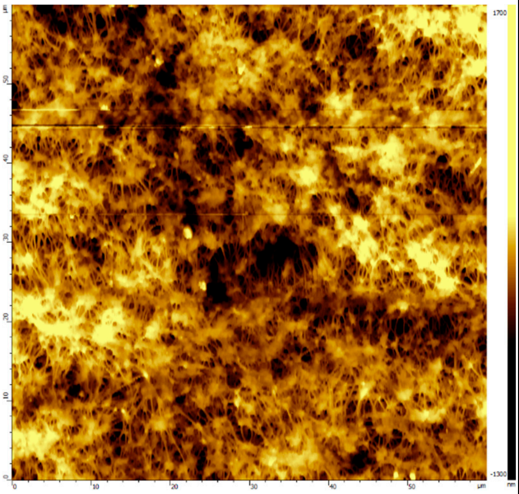	3,0	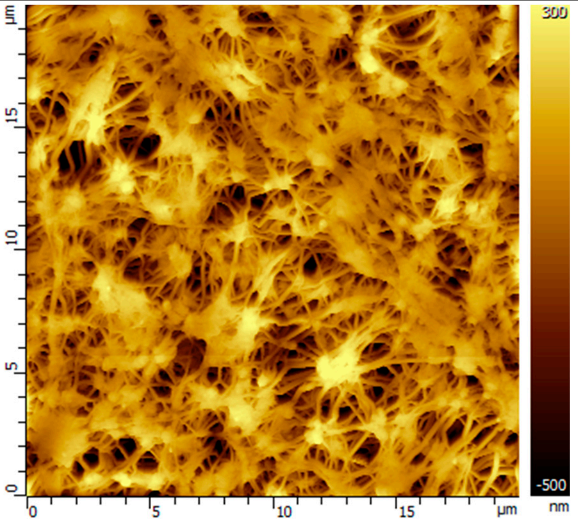	0,8

**Na_2SO_3**	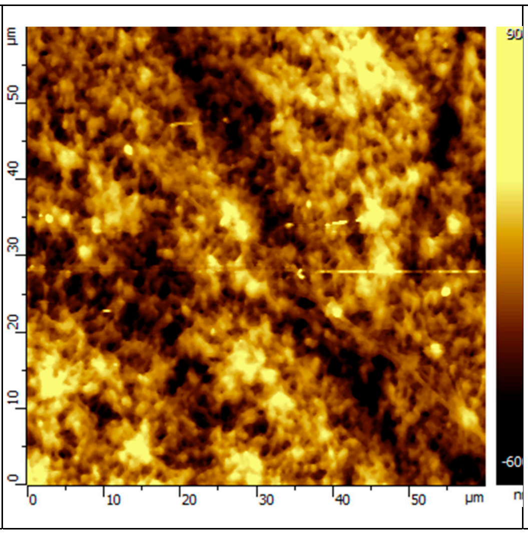	1,5	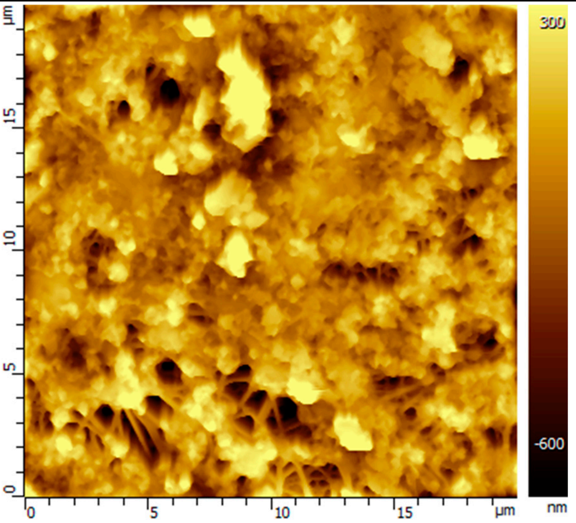	0,9

Table S3. AFM images of PP membranes before and after exposure to model solutions.

**Solution**	**20×20 µm**	**ΔZ, µm**	**10×10 µm**	**ΔZ, µm**
**Initial**	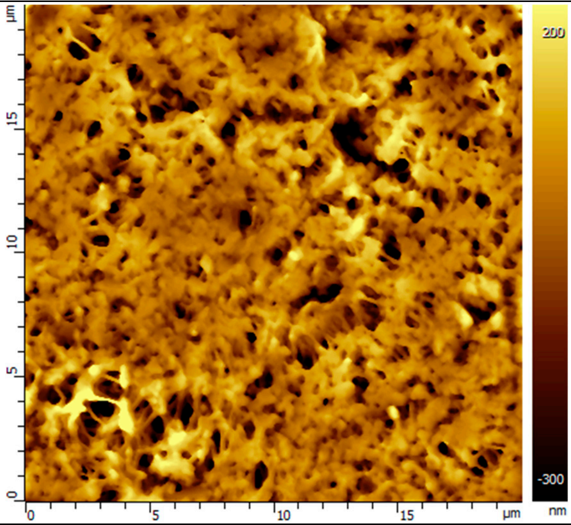	0,5	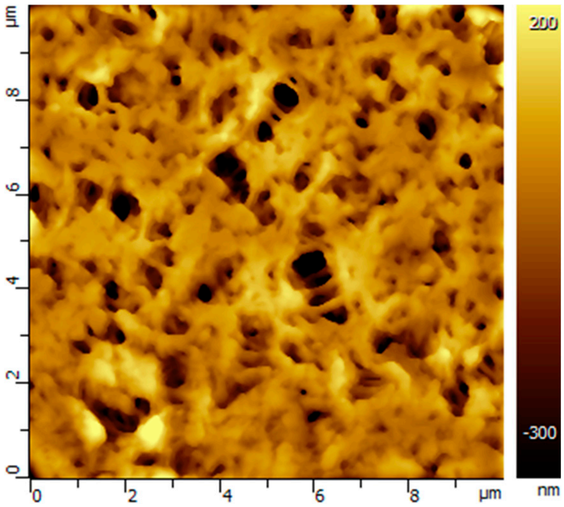	0,5
**MEA**	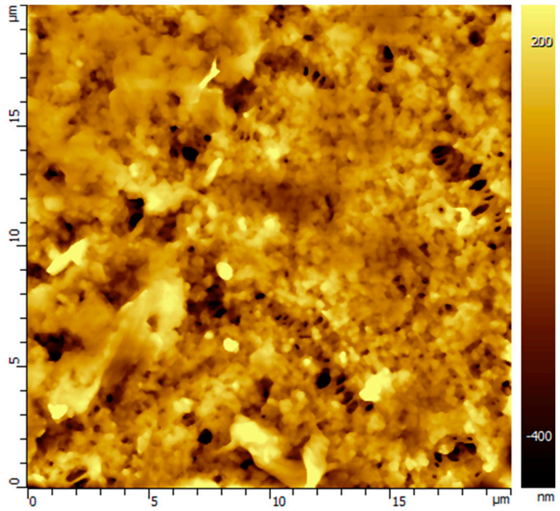	0,6	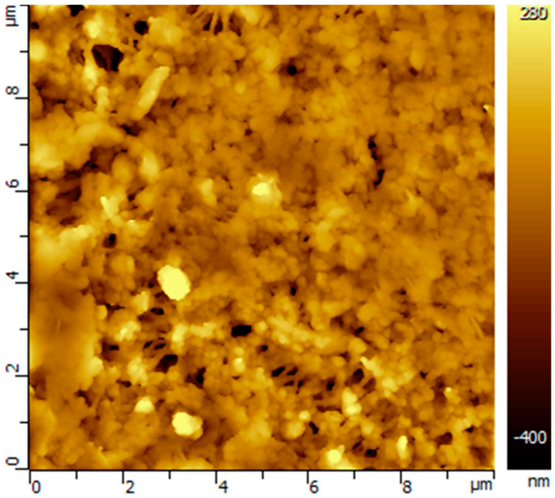	0,68

**DEA**	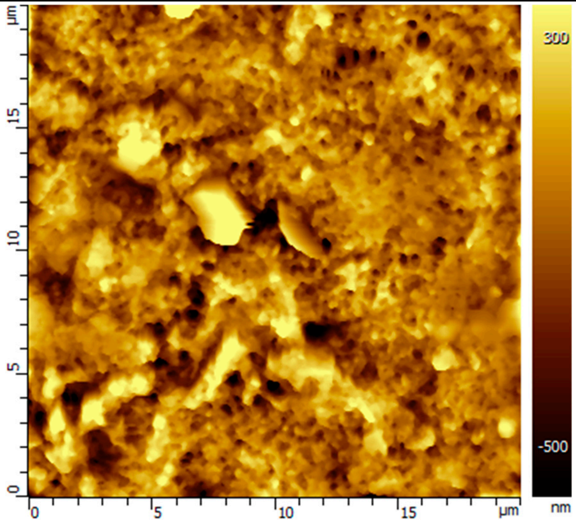	0,8	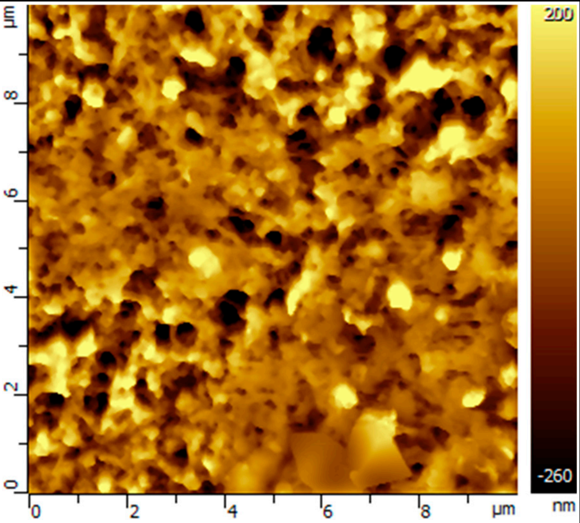	0,46
**MDEA**	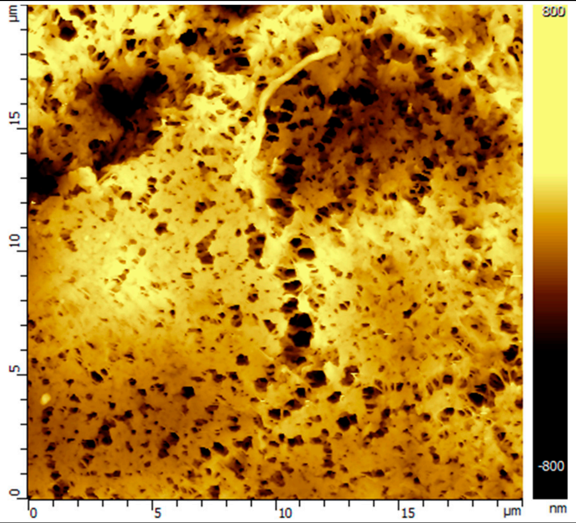	1,6	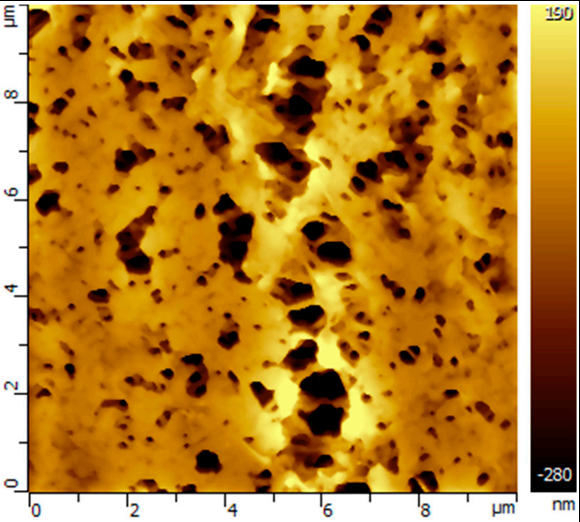	0,47
**AMP**	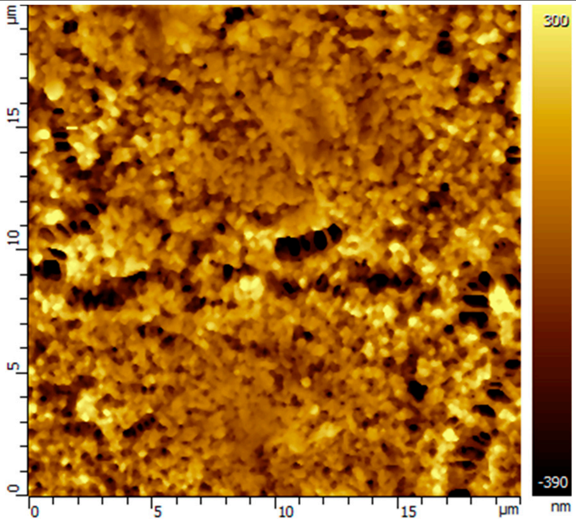	0,69	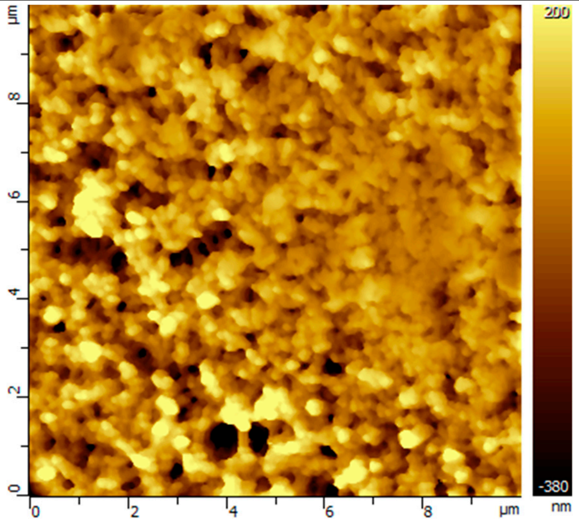	0,58

**PZ**	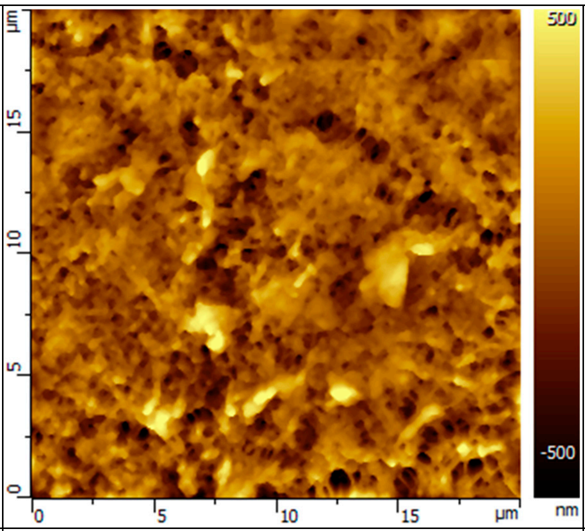	1,0	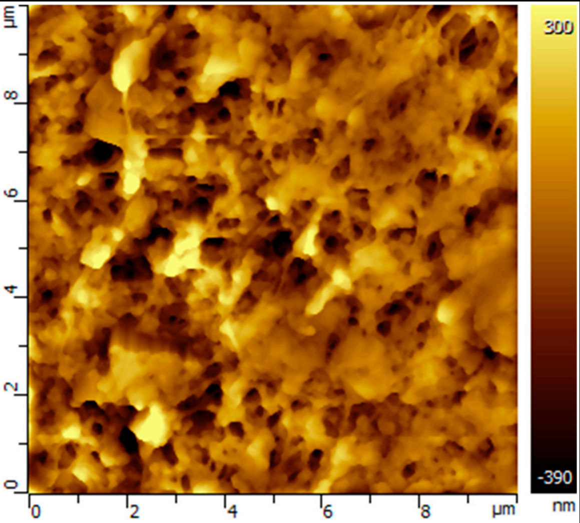	0,69
**Na_2SO_3**	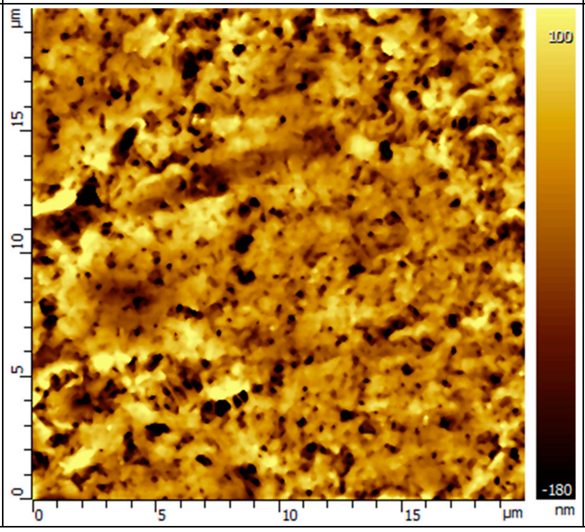	0,28	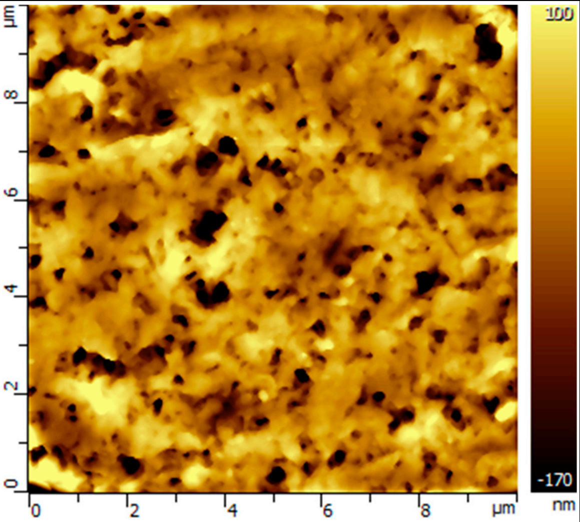	0,27

Table S4. AFM images of PES membranes before and after exposure to model solutions.

**Solution**	**20×20 µm**	**ΔZ, µm**	**10×10 µm**	**ΔZ, µm**
**Initial**	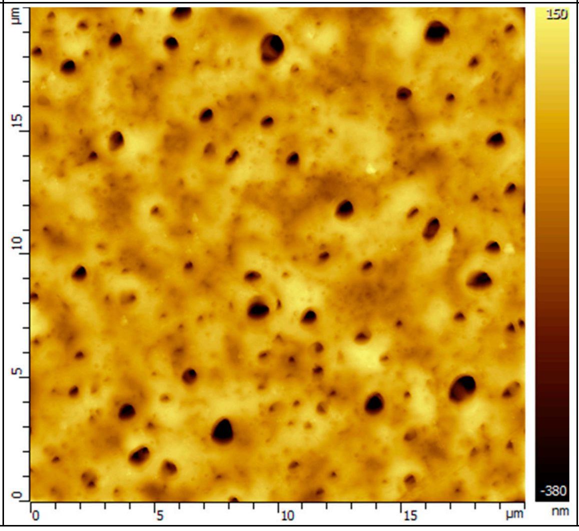	0,53	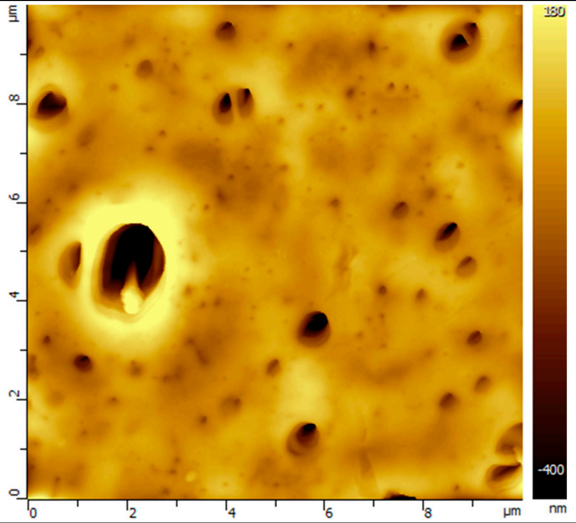	0,58

**MEA**	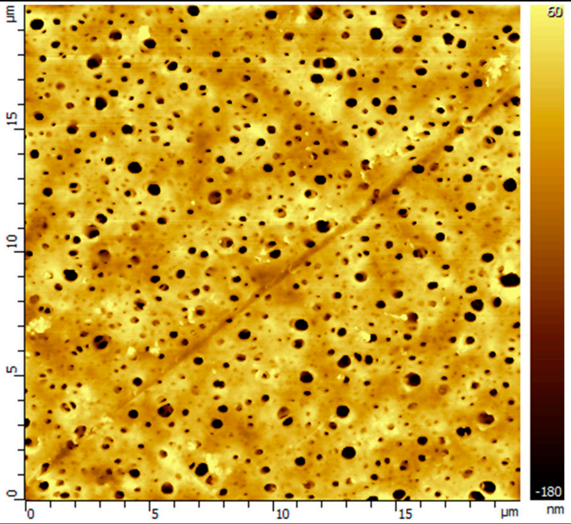	0,24	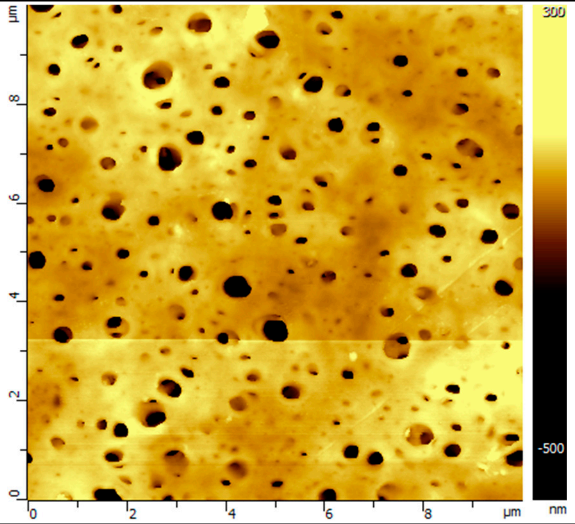	0,8
**DEA**	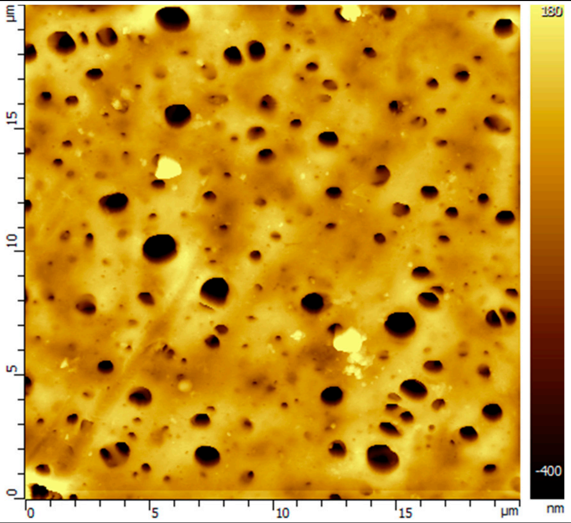	0,58	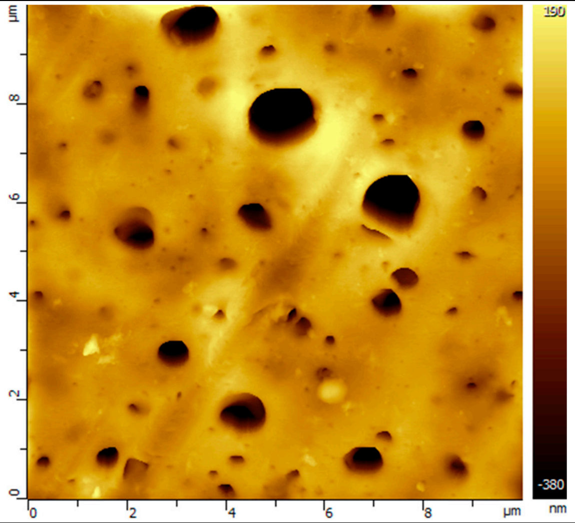	0,57
**MDEA**	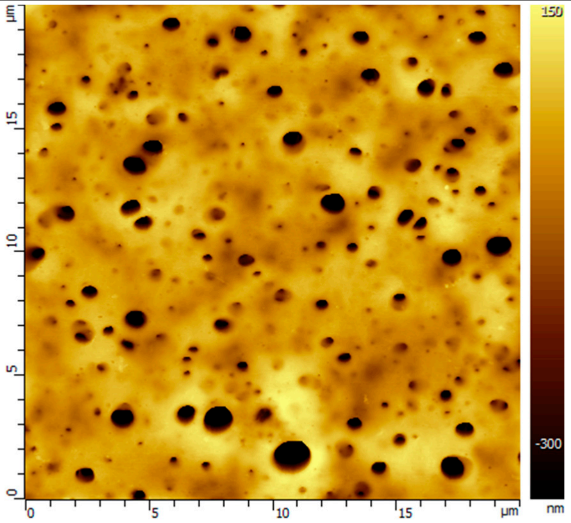	0,45	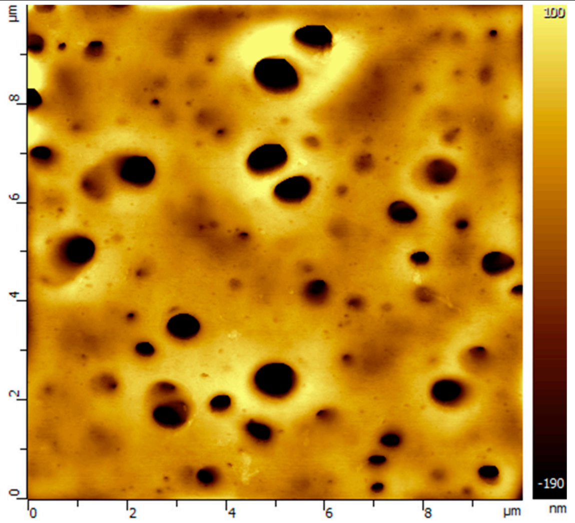	0,29

**AMP**	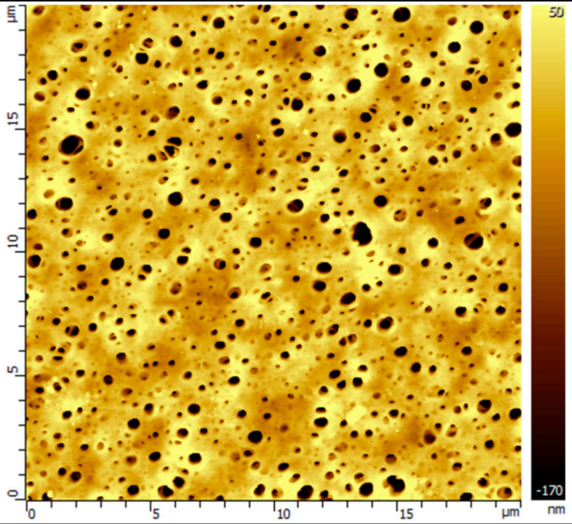	0,22	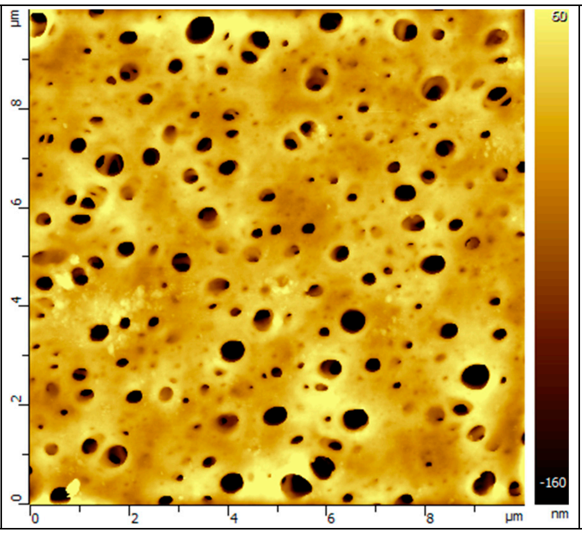	0,22
**PZ**	-			
**Na2SO3**	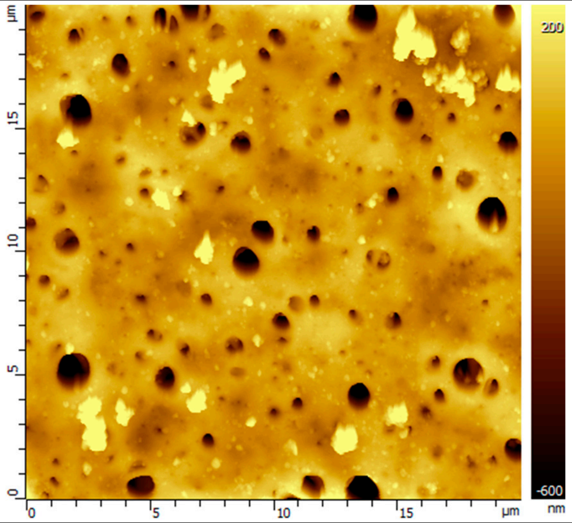	0,8	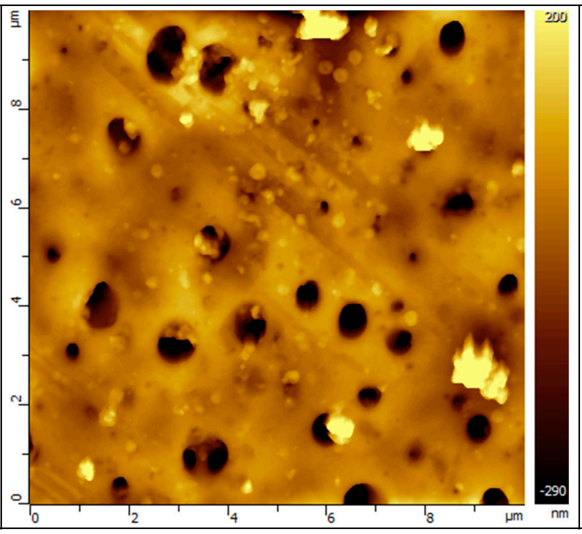	0,49

Table S5. AFM images of PA membranes before and after exposure in model solutions.

**Solution**	**40×40 µm**	**ΔZ, µm**	**20×20 µm**	**ΔZ, µm**
**Initial**	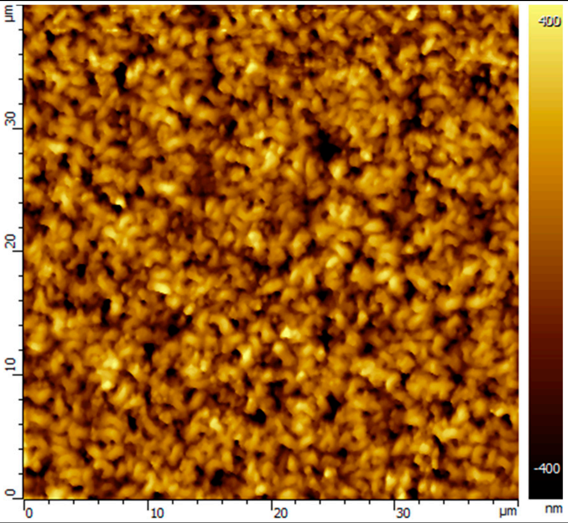	0,8	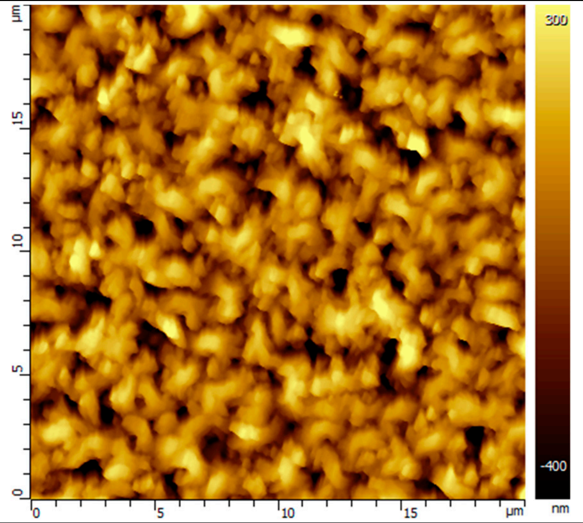	0,7
**MEA**	-			

**DEA**	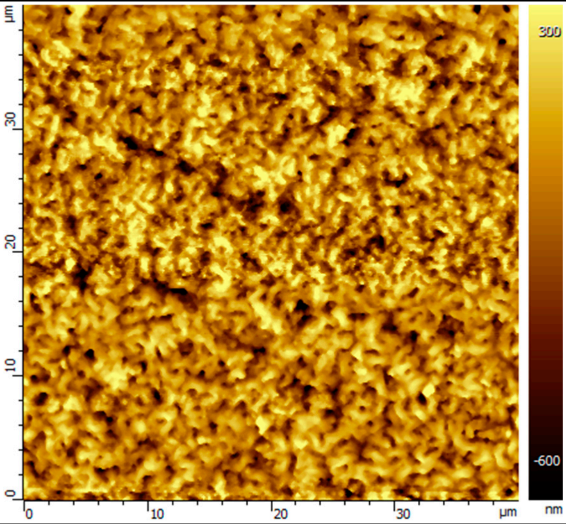	0,9	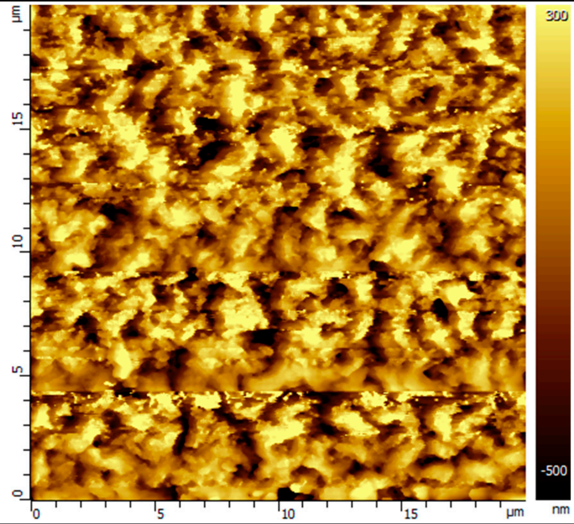	0,8
**MDEA**	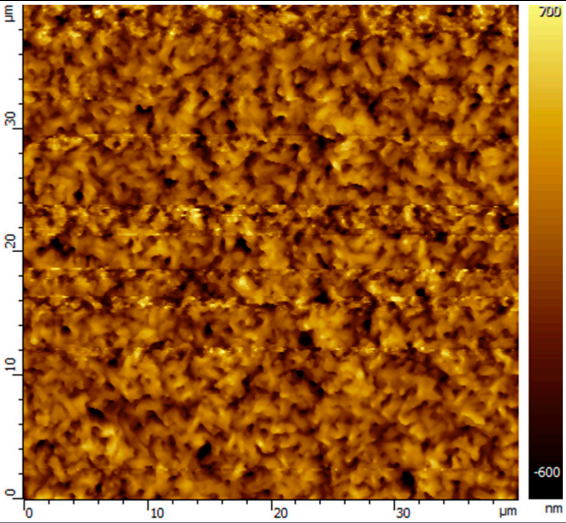	1,3	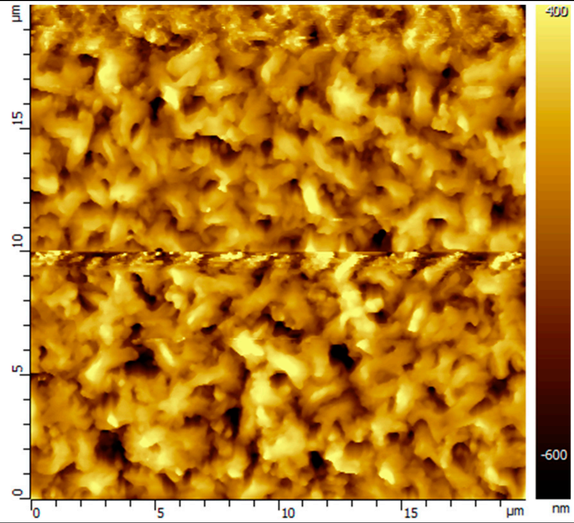	1,0
**AMP**	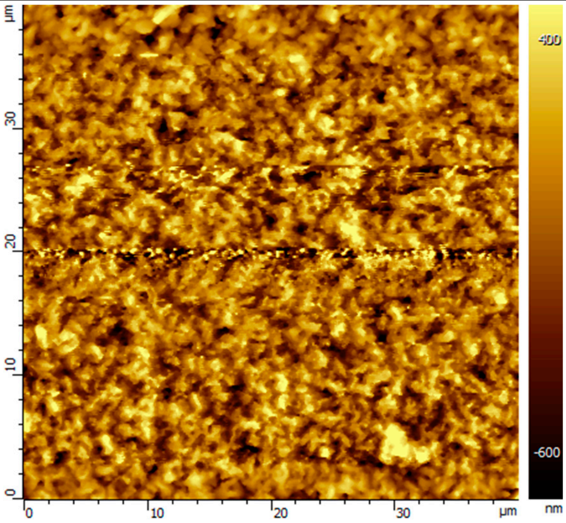	1,0	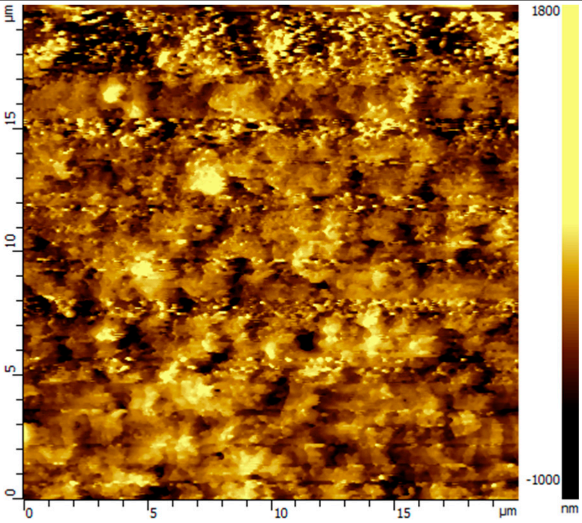	2,8

**PZ**	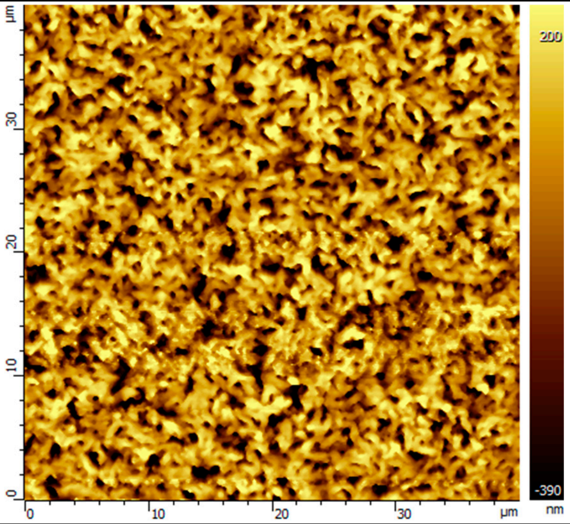	0,59	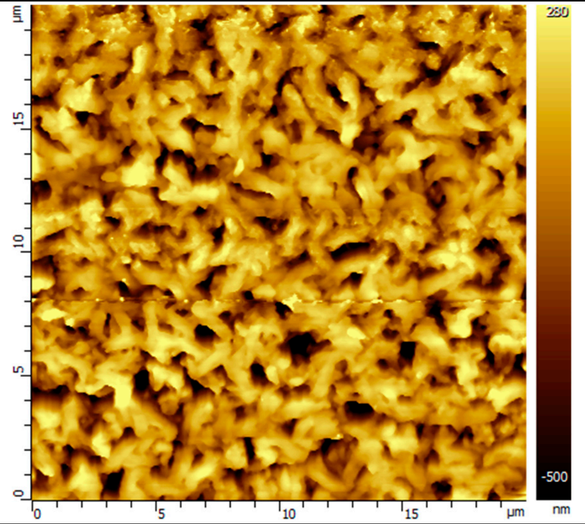	0,78
**Na_2SO_3**	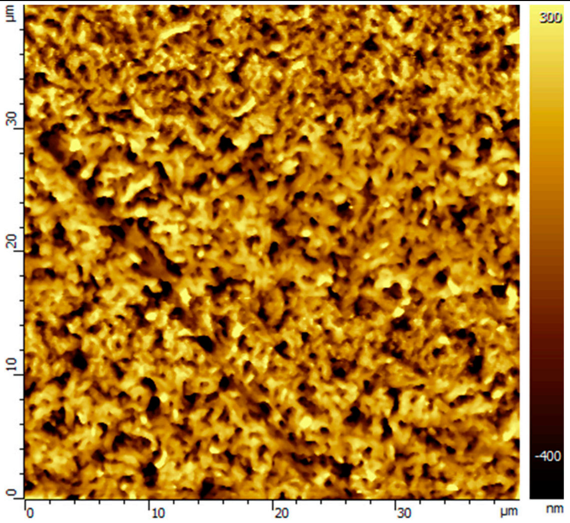	0,7	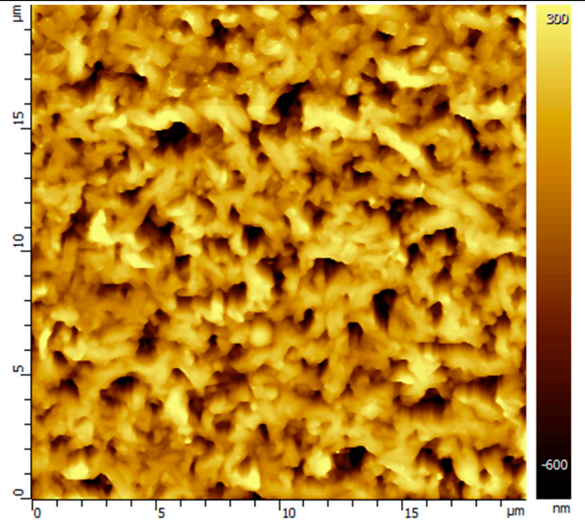	0,9

Table 6. Pore size of the used membranes before and after exposure to degraded amine solvents.

Membrane *	Solution	d_{min}, µm	d_{MFP}, µm	d_{max}, µm
PVDF-022 (PVDF)	Initial	0.40 ± 0.01	0.70 ± 0.01	1.00 ± 0.01
	DEA	0.23 ± 0.01	0.56 ± 0.01	0.85 ± 0.01
MFF4-020 (PTFE)	Initial	0.27 ± 0.01	0.43 ± 0.01	0.56 ± 0.01
	MEA	0.22 ± 0.01	0.43 ± 0.01	0.95 ± 0.01
	DEA	0.32 ± 0.01	0.43 ± 0.01	0.53 ± 0.01
	MDEA	0.32 ± 0.01	0.43 ± 0.01	0.53 ± 0.01
	AMP	0.36 ± 0.01	0.45 ± 0.01	0.53 ± 0.01
	PZ	0.34 ± 0.01	0.44 ± 0.01	0.54 ± 0.01
MCM-010 (Nylon PA)	Initial	0.20 ± 0.01	0.47 ± 0.01	0.53 ± 0.01
PES-020 (PES)	Initial	0.42 ± 0.01	0.72 ± 0.01	0.76 ± 0.01
	MDEA	0.51 ± 0.01	1.06 ± 0.01	1.57 ± 0.01
	AMP	0.56 ± 0.01	0.74 ± 0.01	0.92 ± 0.01
	PZ	0.12 ± 0.01	0.34 ± 0.01	0.57 ± 0.01

* Samples not listed in the table were destroyed after exposure to amines or during measurements due to exposure to pressure.

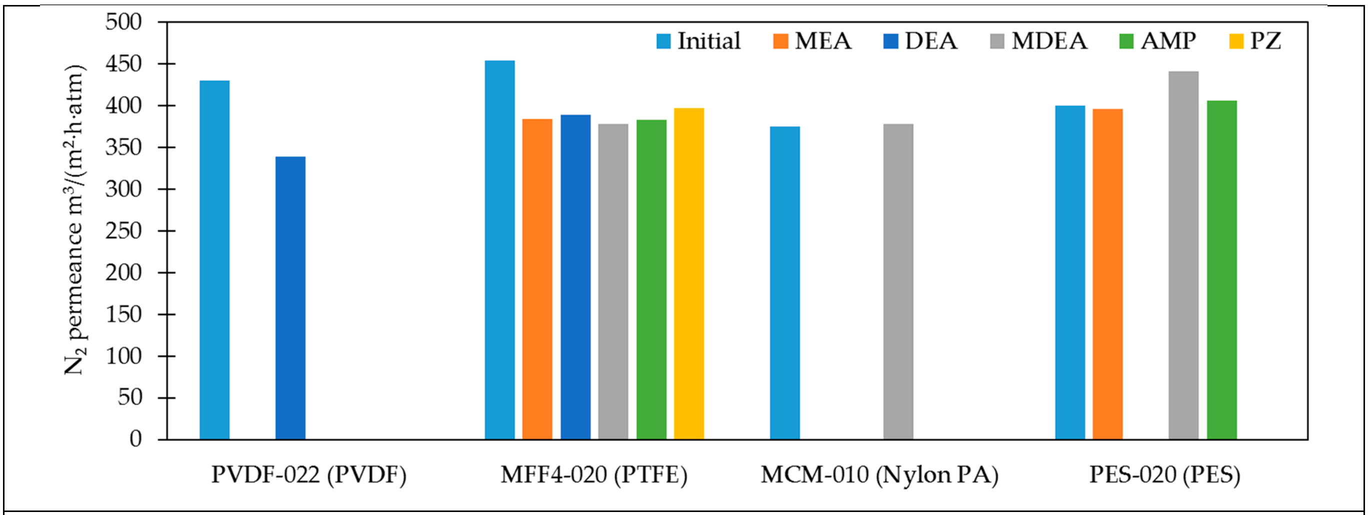

(a)

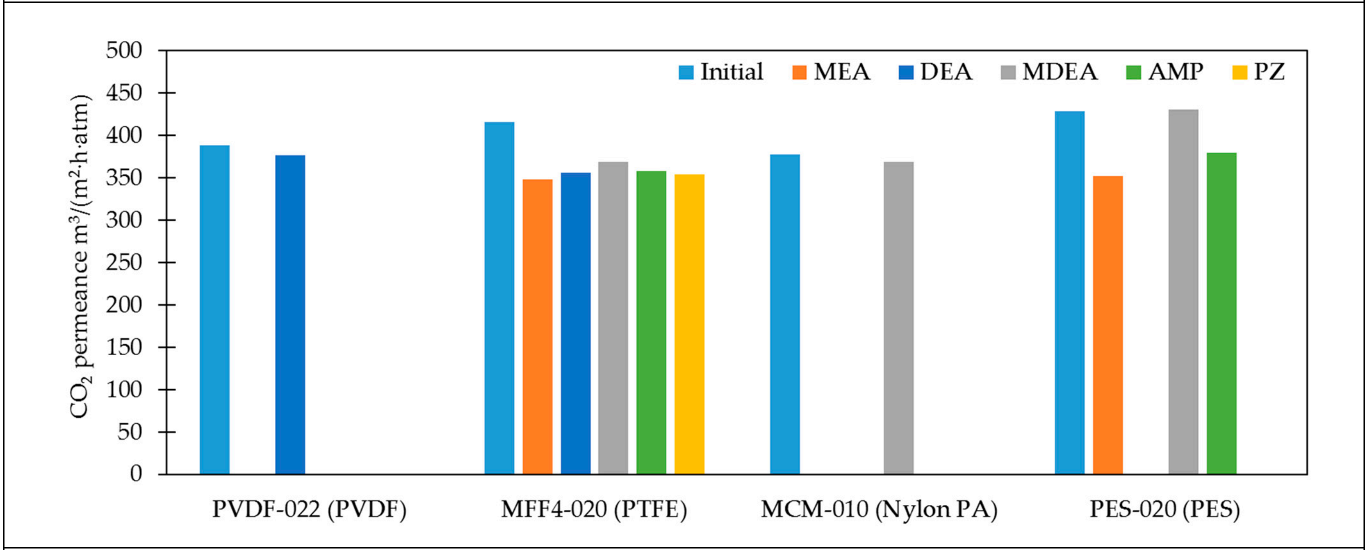

(b)

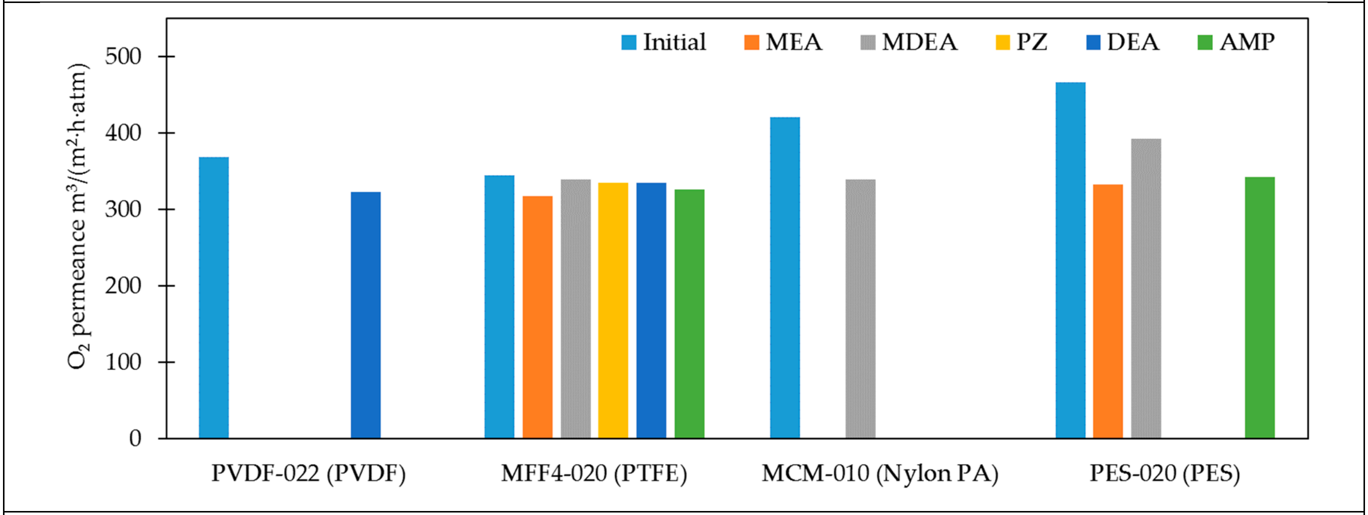

(c)

Figure S4. The permeance of the polymer membranes before and after exposure to model solutions for N_2 (**a**), CO_2 (**b**) and O_2 (**c**).

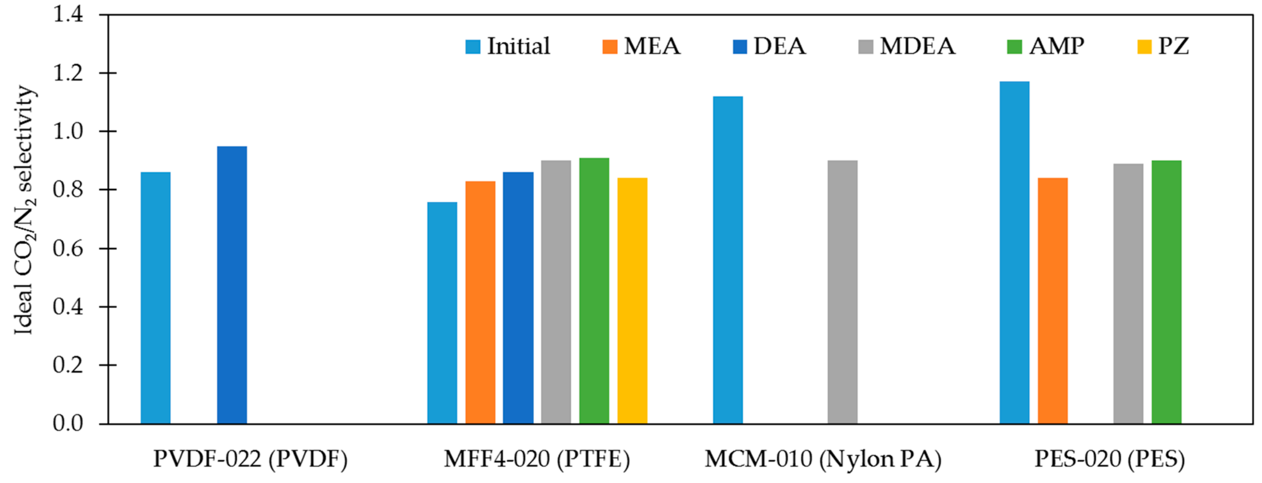

(a)

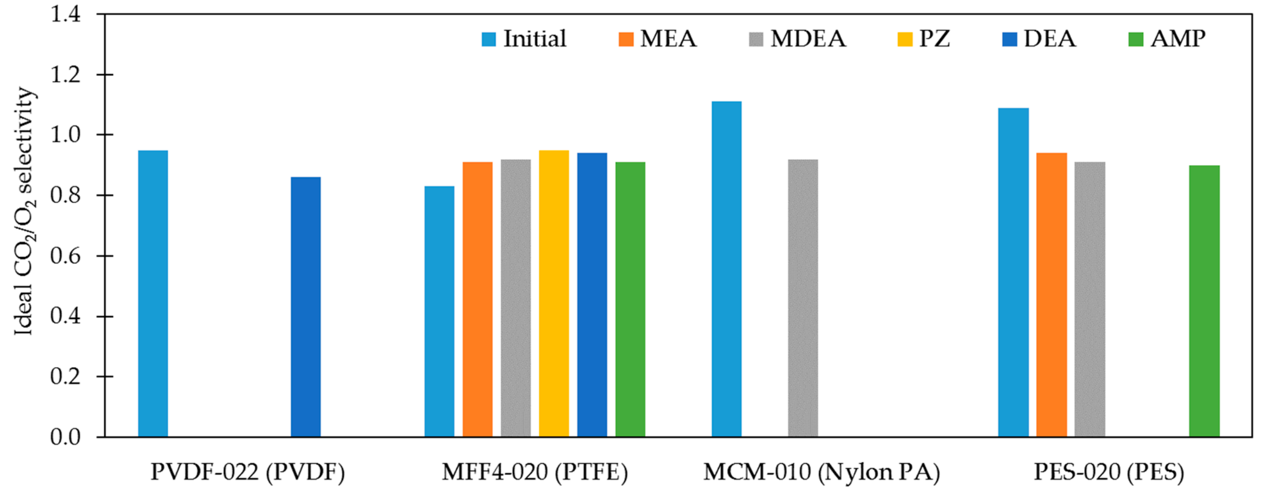

(b)

Figure S5. The ideal selectivity of the polymer membranes before and after exposure to model solutions for CO_2/N_2 (**a**) and CO_2/O_2 (**b**) gas pair.
